# Simulation of Nonisothermal Consolidation of Saturated Soils Based on a Thermodynamic Model

**DOI:** 10.1155/2013/192163

**Published:** 2013-07-31

**Authors:** Zhichao Zhang, Xiaohui Cheng

**Affiliations:** Department of Civil Engineering, Tsinghua University, Beijing 100084, China

## Abstract

Based on the nonequilibrium thermodynamics, a thermo-hydro-mechanical coupling model for saturated soils is established, including a constitutive model without such concepts as yield surface and flow rule. An elastic potential energy density function is defined to derive a hyperelastic relation among the effective stress, the elastic strain, and the dry density. The classical linear non-equilibrium thermodynamic theory is employed to quantitatively describe the unrecoverable energy processes like the nonelastic deformation development in materials by the concepts of dissipative force and dissipative flow. In particular the granular fluctuation, which represents the kinetic energy fluctuation and elastic potential energy fluctuation at particulate scale caused by the irregular mutual movement between particles, is introduced in the model and described by the concept of granular entropy. Using this model, the nonisothermal consolidation of saturated clays under cyclic thermal loadings is simulated in this paper to validate the model. The results show that the nonisothermal consolidation is heavily OCR dependent and unrecoverable.

## 1. Introduction

 Since the 80s of last century, the thermo-hydro-mechanical (THM) coupling problem has become an important scientific research focus in many engineering areas such as geothermal resources development, oil exploration, and nuclear waste storage. Researches show that many physical properties such as the permeability, water content, and thermal expansion coefficient of soils are affected by the temperature [1, 2]. Moreover, the water flow in soils, the pore pressure development, and the shear strength are all sensitive to the temperature variation [3–5]. For example, under temperature elevation, significant thermal volumetric deformation (i.e., the nonisothermal consolidation) [5–7] will occur and the shear strength will be changed significantly. Experimental studies show that the nonisothermal consolidation is heavily dependent on the overconsolidation ratio (OCR) [5–7]. During undrained heating process, due to the difference in the thermal expansion coefficients of solid and fluid phases and the nonelastic deformation, the pore pressure of soils will be increased notably so that thermal failure may occur because of the decrease of effective stress [8]. 

 However, there are few models that can represent all of the THM coupling features discussed before for saturated soils. The existing models at present are usually based on the improvement of the classical elasto-plastic model [8–10] or based on empirical formulae of the pore pressure and strain from the fitting of experimental data [11]. These models are phenomenological and cannot represent the real physical mechanisms of the THM coupling processes in soils. On the contrary, models based on the thermodynamics are able to provide an effective and comprehensive approach for complex multifield coupling problems of soils. In this paper, a thermodynamic theoretical framework, which is usually referred to as hydrodynamic method, is adopted to establish a THM coupling model. In this approach, based on the conservation laws and the nonequilibrium thermodynamics, many independent thermodynamic state variables and thermodynamic potential functions (e.g., free energy) are defined and thus many useful thermodynamic identities that provide a plenty of relations between state variables are obtained theoretically. On the other hand, the unrecoverable energy processes in materials are described by the migration coefficient model in linear nonequilibrium thermodynamics. This approach has been successfully applied in the researches of many different materials such as fluid, crystal, and dry sand [12–16]. Jiang and Liu [14–16] further introduce the concept of granular entropy *S*_*g*_ for granular solid materials to describe the energy process of granular fluctuation, which represents the irregular mutual movement between particles, and derives a constitutive model without the concepts of yield surface, flow rule, and hardening/softening rule.

 Based on the thermodynamic theoretical framework discussed before, a THM coupling model is established for the simulation of nonisothermal consolidation of saturated soils. The granular entropy is introduced to describe the granular fluctuation, which includes the kinetic energy fluctuation and the elastic potential energy fluctuation. A modified form of the evolution law of granular fluctuation is given from the perspective of the physical mechanism of nonisothermal processes, in which the conversion between the free pore water and the bound pore water is considered. Moreover, an equivalent nonelastic strain is defined to determine the elastic potential energy fluctuation and better represent the nonisothermal consolidation under repeated thermal loadings. Based on this model, the nonisothermal consolidation tests are simulated to validate the model.

## 2. Model Formulation

### 2.1. General Assumptions

(1)   Saturated soils can be divided into a solid phase and a liquid phase. The liquid phase is composed of a free water phase and a bound water phase. The bound water can be converted to the free water during temperature elevation, while there is no mass exchange between the solid phase and the liquid phase.
(2) Every phase is continuous in space and all phases have the same temperature.
(3) The liquid phase does not undergo solidification and vaporization in the temperature range considered in this paper, and the soils always maintain saturation.

### 2.2. Basic Equations

 Denote the porosities of the free water and the bound water as *ϕ*_fw_ and *ϕ*_bw_, respectively. The total porosity is *ϕ* = *ϕ*_fw_ + *ϕ*_bw_. Define the density, the velocity, the momentum density, and the entropy density of each phase as *ρ*_*α*_, *v*_*i*_^*α*^, *m*_*i*_^*α*^,  and *s*_*α*_, respectively, wherein, *α* = *s* and *f*, respectively, represent the solid phase, the free water phase and the bound water phase, the same hereinafter. Assume that the bound water is completely absorbed on the surface of the solid particles and thus its velocity equals the velocity of the solid phase; that is, *v*_*i*_^*s*^ = *v*_*i*_^bw^.

#### 2.2.1. Mass Conservation Equations

 The mass conservation equations for the solid phase, the free water phase, and the bound water phase are shown in (1a)–(1c), respectively, wherein, *d*_*t*_ is the material derivative using the solid phase as a reference and has a relation with the spatial derivative ∂_*t*_, *d*_*t*_ = ∂_*t*_ + *v*_*k*_^*s*^∇_*k*_. The material derivative is used in this paper for the simplification of the forms of partial differential equations. *v*_*i*_^fw^ −  *v*_*i*_^*s*^ is the relative velocity of the free water and the solid phase, and thus *ϕ*_fw_(*v*_*k*_^fw^ − *v*_*k*_^*s*^)  is just the average water flow velocity. *ε*_*kk*_ is the volumetric strain of the solid skeleton and is taken as positive under compression. The assumption of small strain is adopted; that is, the strain rate *d*_*t*_ *ε*_*ij*_ = −(*v*_*i*,*j*_^*s*^ + *v*_*j*,*i*_^*s*^)/2.  *Q* represents the mass converted from bound water to free water per unit time and per unit volume during the elevation of temperature. In this paper, *Q* = *ρ*_bw_ *α*_bf_ *ϕ*_bw_ *d*_*t*_ *T*, wherein, the parameter *α*_bf_ is the mass of free water converted from per unit mass of bound water per each unit rise in temperature:

(1a) $$\begin{array}{c} d_{t}\left[\rho_{s}\left(1-\phi \right)\right]=\rho_{s}\left(1-\phi \right)d_{t}\varepsilon_{kk}, \end{array}$$

(1b) dt(ρfwϕfw)=−ρfw[ϕfw(vkfw−vks)],k −ϕfw(vkfw−vks)∇kρfw +ρfwϕfwdtεkk+Q,

(1c) $$\begin{array}{c} d_{t}\left(\rho_{\text{bw}}\phi_{\text{bw}}\right)=\rho_{\text{bw}}\phi_{\text{bw}}d_{t}\varepsilon_{kk}-Q. \end{array}$$

The average water flow velocity can be determined by a generalized flow formula [10, 17] as shown in (2), where *k* is the intrinsic permeability of the solid skeleton, *μ* is the kinematic viscosity of the fluid, *g*_*i*_ is the gravity acceleration vector, and *T* is the temperature. The term *θ*_*ik*_∇_*k*_ *T* in (2) represents the coupling between the water flow and the thermal conduction, where *θ*_*ik*_ is a thermal coupling coefficient [10]:

(2) $$\begin{array}{c} \phi_{\text{fw}}\left(v_{i}^{\text{fw}}-v_{i}^{s}\right)=-\frac{k}{\mu }\left[\nabla_{i}p-\rho_{\text{fw}}g_{i}\right]-\theta_{ik}\nabla_{k}T. \end{array}$$

In (1a), (1b), (1c), and (2), the density of each phase, the intrinsic permeability of the solid skeleton, and the kinematic viscosity of the fluid are the functions of temperature and stresses, as shown in (3a), (3b), (4a), and (4b). Wherein, *p*′ is the effective mean stress, *p* is the pore pressure, *K*_*s*_ is the bulk modulus of the solid particle, *c*_*f*_ is the compressibility of the liquid phase, and *T*_0_ is the reference temperature. *ρ*_*s*0_ and *ρ*_*f*0_ are the densities of solid and liquid phases, respectively, at zero pressure and the temperature *T* = *T*_0_. As shown in (4a), *k* is the function of the porosity, where *k*_0_ and *b*_*k*_ are material parameters. The kinematic viscosity of the fluid is the function of temperature [18], as in (4b):

(3a) $$\begin{array}{c} \rho_{s}=\rho_{s0}\left[1-3\beta_{s}\left(T-T_{0}\right)+\frac{p^{\prime }}{\left(1-\phi \right)K_{s}}\right], \end{array}$$

(3b) $$\begin{array}{c} \rho_{\text{fw}}=\rho_{\text{bw}}=\rho_{f0}\left[1+c_{f}p-\beta_{f}\left(T-T_{0}\right)\right], \end{array}$$

(4a) $$\begin{array}{c} k=k_{0}\exp\left(\frac{b_{k}}{1-\phi_{\text{fw}}}\right), \end{array}$$

(4b) $$\begin{array}{c} \mu =1.984\times 10^{-6}\exp\left(\frac{1825.85}{T}\right)\left(\text{N}\cdot \text{s}/{\text{m}}^{2}\right). \end{array}$$

#### 2.2.2. Momentum Conservation Equation

 The momentum conservation for saturated soils is shown in (5). Wherein,  *σ*_*ij*_^*s*^,  *σ*_*ij*_^fw^,  and *σ*_*ij*_^bw^ are the stresses of the solid phase, the free water phase, and the bound water phase, respectively. All of them are defined as average stresses on the section with a total surface porosity of *ϕ* and taken as positive under compression. Thus, the pore pressure also contributes to the stress of solid phase. According to the mixture theory [10], stresses of each phase can be expressed as shown in (6a), (6b), and (6c), where *σ*_*ij*_′ is the effective stress and *K*_*b*_ is the bulk modulus of the solid skeleton. Thus, the total stress can be written as in (7), where *α* is the Biot coefficient which takes the compressibility of solid particles into account. Equation (7) is just the generalized effective stress principle in soil mechanics:

(5) ρs(1−ϕ)dtvis+ρbwϕbwdtvibw   +ρfwϕfwdtvifw+(vifw−vis)ρfwϕfw∇jvifw   +∇j(σijs+σijfw+σijbw)  =gi[ρs(1−ϕ)+ρfwϕfw+ρbwϕbw],

(6a) $$\begin{array}{c} \sigma_{ij}^{s}=\sigma_{ij}^{\prime }+\left[1-\frac{K_{b}}{K_{m}\left(1-\phi \right)}\right]p\left(1-\phi \right)\delta_{ij}, \end{array}$$

(6b) $$\begin{array}{c} \sigma_{ij}^{\text{fw}}=p\phi_{\text{fw}}\delta_{ij},\; \end{array}$$

(6c) $$\begin{array}{c} \sigma_{ij}^{\text{bw}}=p\phi_{\text{bw}}\delta_{ij}, \end{array}$$

(7) $$\begin{array}{c} \sigma_{ij}=\sigma_{ij}^{s}+\sigma_{ij}^{\text{fw}}+\sigma_{ij}^{\text{bw}}=\sigma_{ij}^{\prime }+\alpha p\delta_{ij},\;\alpha =1-\frac{K_{b}}{K_{m}}. \end{array}$$

#### 2.2.3. Entropy Increase Equation

 In the thermodynamic framework of the hydrodynamic method [19, 20], the energy conservation laws are fully used in the derivation of the constitutive relations. Therefore, the entropy increase equation is adopted as the governing equation of the thermal field. According to the thermodynamics, the change of entropy should consist of the entropy production and the entropy flow induced by water flow, thermal conduction, and thermal convection. Define the specific entropy of the solid phase, the free water phase, and the bound water phase as *υ*_*s*_, *υ*_fw_, and *υ*_bw_, respectively. The entropy increase equation of saturated soils is

(8a) ρs(1−ϕ)dtυs+ρfwϕfwdtυfw   +ρbwϕbwdtυbw=RT+∇kκ∇kTT   −(vkfw−vks)ρfwϕfw∇kυfw,

(8b) $$\begin{array}{c} \upsilon_{s}=C_{s}\ln\left(\frac{T}{T_{0}}\right)+\upsilon_{s0}, \end{array}$$

(8c) $$\begin{array}{c} \upsilon_{\text{fw}}=\upsilon_{\text{bw}}=C_{f}\ln\left(\frac{T}{T_{0}}\right)+\upsilon_{f0}, \end{array}$$

where *C*_*s*_ and *C*_*f*_ are the specific heat capacity of solid and fluid phases, respectively; *υ*_*s*0_ and *υ*_*f*0_ are the specific entropy of solid and fluid phases, respectively, at  *T* = *T*_0_; *κ* is the effective thermal conductivity of saturated soils; *R* is the energy dissipation rate induced by all unrecoverable process, including the viscosity of each phase, the thermal conduction, the pore water flow, the transient elasticity, and the granular fluctuation [14–16, 21]. *R*/*T* is called entropy production rate. In this paper, only the dissipations corresponding to the transient elasticity and the granular fluctuation are considered for the purpose of simplification, as they are the most important dissipation mechanisms for saturated soils. 

The transient elasticity, which corresponds to the relaxation of elastic potential energy and means that the materials are no longer elastic as long as external loadings begin, is an important mechanism of nonelastic deformation of solid materials [14–16]. For granular solid materials, under external loadings, there are random fluctuation movements at the granular scale around the macroaverage movements, such as the slide, roll, and collision between particles, corresponding to the kinetic energy fluctuation. Along with the kinetic energy fluctuation, there is also an elastic potential energy fluctuation at granular scale since that the elastic potential energy is stored through the interactions between particles. The granular fluctuation is always accompanied by the energy dissipation and is the important source of nonelastic deformation for granular solid materials. If there are no continuous incentives, the granular fluctuation will be attenuated by the form of macroenergy dissipation (i.e., heat generation) until the fluctuation disappears. Unlike the discrete approach in granular mechanics, this paper adopts a continuum method called “double entropy” theory proposed by Jiang and Liu [14–16] who introduce the concepts of granular entropy density *s*_*g*_ and the granular entropy temperature *T*_*g*_ to give a unified consideration of the granular fluctuation.

According to the nonequilibrium thermodynamics [19, 20], energy dissipation rate *R* can be expressed as the sum of multiplying all dissipative forces (generalized force that represents the driving actions making a system deviate from the thermodynamic equilibrium state) and corresponding dissipative flows. The dissipative forces of transient elasticity and granular fluctuation are the thermodynamic conjugate state variable of the elastic strain and the granular entropy temperature [16, 21], denoted as *π*_*ij*_ and *T*_*g*_, respectively. *T*_*g*_ is the conjugate variable of the granular entropy density *s*_*g*_ and presents the severe degree of the granular fluctuation. It can be proved that *π*_*ij*_ approximatively equals the effective stress of saturated soils; that is, *π*_*ij*_ ≈ *σ*_*ij*_′ [21]. Define the dissipative flows corresponding to *π*_*ij*_  and *T*_*g*_ as *Y*_*ij*_ and *I*_*g*_, respectively. Thus,

(9) $$\begin{array}{c} R=Y_{ij}\pi_{ij}+I_{g}T_{g}. \end{array}$$

According to the linear nonequilibrium thermodynamics, the dissipative flows can be expressed as the functions of dissipative forces, as in (10a) and (10b). Wherein, *λ*_*ijkl*_ and *γ* are called migration coefficients:

(10a) $$\begin{array}{c} Y_{ij}=\lambda_{ijkl}\pi_{kl}, \end{array}$$

(10b) $$\begin{array}{c} I_{g}=\gamma T_{g}. \end{array}$$

### 2.3. Constitutive Relations of Mechanical Field

 As long as evolution laws of the strain of solid skeleton and the effective stress are determined, a completed THM coupling model for saturated soils can be established, using (1a)–(1c)–(10a) and (10b). It is worthwhile mentioning that in the framework of hydrodynamic method [12–16], all of these evolution laws can be derived theoretically. The derivation process of these laws can be found in [16], and the results are given directly in this paper.

#### 2.3.1. Hyperelastic Relation

 In this paper, the effective stress is defined as the function of elastic strain *ε*_*ij*_^*e*^ of the solid skeleton through the elastic potential energy density function *ω*_*e*_. This hyperelastic relation can be expressed as

(11) $$\begin{array}{c} \sigma_{ij}^{\prime }\approx \pi_{ij}=\frac{\partial \omega_{e}}{\partial \varepsilon_{ij}^{e}}. \end{array}$$

Introducing a nonlinear term to the linear model of *ω*_*e*_, Jiang and Liu [14–16] proposed a model for dry sands that can represent the effect of effective mean stress on the elastic modulus and provide a maximum stress ratio in effective stress space. Based on the Jiang and Liu model, this paper proposes a new model of *ω*_*e*_ as shown in (12a) and (12b), considering the cohesion of soils and the thermoelastic coupling:

(12a) ωe=25B(εve+c)1.5(εve)2+Bξ(εve+c′)1.5(εse)2 +∫3KeβT(T−T0)dεve,

(12b) $$\begin{array}{c} B=B_{0}\exp\left(B_{1}\rho_{d}\right),{\;\varepsilon }_{v}^{e}=\varepsilon_{kk}^{e},\;\varepsilon_{s}^{e}=\sqrt{e_{ij}^{e}e_{ij}^{e}}. \end{array}$$

In (12a) and (12b), *ε*_*v*_^*e*^ and *ε*_*s*_^*e*^ are the elastic volumetric strain and the second invariant of the elastic strain; *e*_*ij*_ = *ε*_*ij*_ − *ε*_*kk*_ *δ*_*ij*_/3 is the deviatoric strain tensor; *B*_0_ is a parameter with the same dimension as stresses; *ρ*_*d*_ is the dry density of the saturated soils, that is, *ρ*_*d*_ = *ρ*_*s*_(1 − *ϕ*); *B*_1_ is a parameter that represents the effect of the dry density on the elastic modulus and shear strength of soils; *c* is a parameter related to the cohesion and should be taken as zero for cohesionless materials like sands; *ξ* is a parameter related to the shear behavior of soils; *c*′ is a parameter relevant to the critical shear strength. The last term in (12a) and (12b) is the thermoelastic coupling term, where *β*_*T*_ is the elastic expansion coefficient of the solid skeleton and *K*_*e*_ is the secant elastic bulk modulus of the solid skeleton. *K*_*e*_ is defined by *π*_*kk*_ = 3*K*_*e*_[*ε*_*v*_^*e*^ + *β*_*T*_(*T* − *T*_0_)]. Thus, from (11), (12a), and (12b),

(13) Ke=0.6B(εve+c)0.5εve +1.5Bξ(εve+c′)0.5(εse)2εve+0.8B(εve+c)1.5,

provided that *β*_*T*_ is only dependent on the thermal expansion of the solid phase and the bound water phase, as the thermal expansion of the free water does not contribute to the elastic deformation of the solid skeleton. Thus,

(14) $$\begin{array}{c} \beta_{T}=\beta_{s}\left(1-\phi \right)+\beta_{f}\phi_{\text{bw}}. \end{array}$$

#### 2.3.2. “Granular Entropy Increase” Equation

Define the specific granular entropy as *υ*_*g*_ = *s*_*g*_/*ρ*_*s*_. Similar to the entropy increase equation, the “granular entropy increase” equation is

(15a) $$\begin{array}{c} \rho_{s}\left(1-\phi \right)d_{t}\upsilon_{g}=\frac{R_{g}}{T_{g}}-I, \end{array}$$

(15b) $$\begin{array}{c} \;R_{g}=\sigma_{ij}^{g}d_{t}\varepsilon_{ij}+Md_{t}T, \end{array}$$

(15c) $$\begin{array}{c} I=\frac{I_{g}T_{g}+Y_{ij}^{g}\pi_{ij}}{T_{g}}, \end{array}$$

(15d) $$\begin{array}{c} \sigma_{ij}^{g}=\eta_{g}T_{g}d_{t}e_{ij}+\zeta_{g}T_{g}d_{t}\varepsilon_{kk}\delta_{ij}, \end{array}$$

(15e) M=ψgTgπkkαbfϕbw3(1−ϕ), (ψg>0  (dtT>0);             ψg=0  (dtT≤0)),

where *R*_*g*_ is the granular fluctuation energy production rate induced by external stimulations and can be determined by (15b) using a similar method described in (9) (*R*_*g*_ ≥ 0). Note that both mechanical loadings and thermal loading can stimulate the reorganized movement of the particles, such as the mechanical consolidation and the nonisothermal consolidation [5–7]. In this paper, the “dissipative forces” for granular fluctuation are the strain rate and the temperature rate, as in (15b). The “dissipative flows” corresponding to *d*_*t*_ *ε*_*ij*_ and *d*_*t*_ *T* are denoted as *σ*_*ij*_^*g*^ and *M*, respectively. *σ*_*ij*_^*g*^ is expressed as a linear function of the strain rate, as in (15d), where *η*_*g*_ and *ζ*_*g*_ are material parameters also called migration coefficients. *M* can be theoretically determined by the conversion process from bound water to free water during temperature elevation [21], as in (15e), where *ψ*_*g*_ is a material parameter. 

Different from the entropy, the granular entropy can be converted to the “real” entropy. In (15a)–(15e), *I* is the conversion rate of the granular entropy to the real entropy and should satisfy *I* ≥ 0. In (15c), *I*_*g*_ *T*_*g*_ and *Y*_*ij*_^*g*^ *π*_*ij*_ are the dissipation rates of the kinetic energy fluctuation and the elastic potential energy fluctuation into real entropy, respectively. *Y*_*ij*_^*g*^ is the elastic potential energy fluctuation “dissipative flow.” The energy dissipation corresponding to the elastic potential energy fluctuation described by *Y*_*ij*_^*g*^ *π*_*ij*_ will be transformed to be a part of the transient elasticity dissipation described by *Y*_*ij*_ *π*_*ij*_ in (9). The energy dissipation rate *I*_*g*_ *T*_*g*_ described in (9) represents only the energy dissipation of granular kinetic energy fluctuation.

By defining a granular fluctuation energy density function, the relation between the specific granular entropy *υ*_*g*_ and the granular entropy temperature *T*_*g*_ can be obtained [16, 21], as shown as follows (16):

(16) $$\begin{array}{c} \upsilon_{g}={bT}_{g}. \end{array}$$

#### 2.3.3. Evolution Laws of Elastic and Nonelastic Strains

 Divide the strain rate into the elastic strain rate *d*_*t*_ *ε*_*ij*_^*e*^ and the nonelastic strain *d*_*t*_ *ε*_*ij*_^*D*^; that is,

(17) $$\begin{array}{c} d_{t}\varepsilon_{ij}^{e}=d_{t}\varepsilon_{ij}-d_{t}\varepsilon_{ij}^{D}. \end{array}$$

In this paper, the nonelastic strain evolution is quantitatively determined by the dissipations induced by the transient elasticity and the granular fluctuation described by (9), (10a), (10b), (15a)–(15e), and (16). Using the thermodynamic principles proposed by Jiang and Liu [16], it can be proved that the nonelastic strain rate has a relation with the dissipative flow *Y*_*ij*_ and *Y*_*ij*_^*g*^, as shown in (18a). Here, a simplified model for the dissipative flow *Y*_*ij*_ described in (10a) has been proposed as the so-called relaxation time model [16, 21], as in (18b). *λ*_*v*_, *λ*_*s*_, and *a* are material parameters. The transient elasticity must be stimulated by the granular fluctuation. Therefore, in (18b), *Y*_*ij*_ = 0 when *T*_*g*_ = 0:

(18a) $$\begin{array}{c} d_{t}\varepsilon_{ij}^{D}=Y_{ij}-Y_{ij}^{g}, \end{array}$$

(18b) $$\begin{array}{c} Y_{ij}=\lambda_{s}{\left(T_{g}\right)}^{a}e_{ij}^{e}+\lambda_{v}{\left(T_{g}\right)}^{a}\varepsilon_{kk}^{e}\delta_{ij}. \end{array}$$

Following with (18b), an equivalent nonelastic strain *ε*_*ij*_^*h*^ is introduced to determine the value of *Y*_*ij*_^*g*^, as in (19a). The evolution of *ε*_*ij*_^*h*^ is defined by the nonelastic strain rate, as shown in (19b). *w* and *h* are material parameters. The second term on the right side of (19b) restricts the value of $\sqrt{\varepsilon_{ij}^{h}\varepsilon_{ij}^{h}}$ within a maximum value *h*:

(19a) $$\begin{array}{c} Y_{ij}^{g}=\lambda_{s}{\left(T_{g}\right)}^{a}e_{ij}^{h}+\lambda_{v}{\left(T_{g}\right)}^{a}\varepsilon_{kk}^{h}\delta_{ij}, \end{array}$$

(19b) dtεijh=dtεijD−wdtεklD·εklhhεmnhεmnh ×εijh {w=1dtεklD·εklh>00<w<1dtεklD·εklh<0.

Setting ${\tilde{T}}_{g}=\lambda_{s}^{1/a}T_{g}$, *m*_1_ = *λ*_*v*_/*λ*_*s*_, *m*_2_ = (*λ*_*s*_)^1/*a*^ *η*_*g*_/*γ*, *m*_3_ = *ζ*_*g*_/*η*_*g*_,  *m*_4_ = *γ*/*b* and *m*_5_ = (*λ*_*s*_)^1/*a*^ *ψ*_*g*_/*b* and combining with (10b), (15a)–(15e), and (16), the “granular entropy increase” equation expressed with *T*_*g*_ can be written in (20). The term *Y*_*ij*_^*g*^ *π*_*ij*_ is ignored in (20) for the simplification of the model calculation and parameter calibration:

(20) dtT~g=m2m4dteijdteij+m2m3m4(dtεkk)2ρd +m5πkkαbfϕfw3ρd(1−ϕ)dtT−m4ρdT~g.

### 2.4. Model Summary

**Figure 1:**
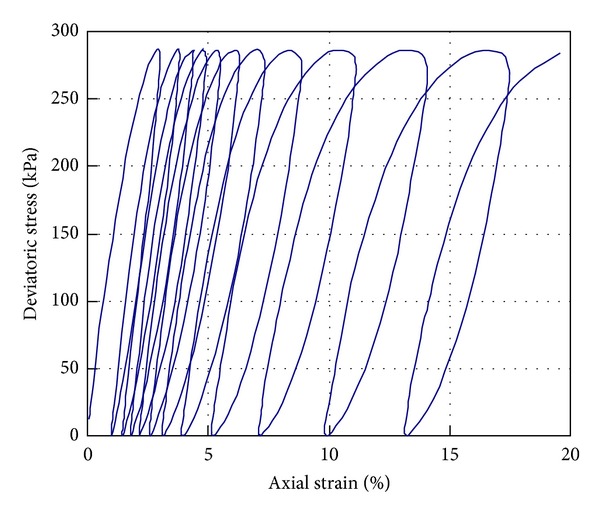
Simulation result of one-way cyclic undrained triaxial shear test using the constitutive model presented in this paper.

 The mass conservation equations (1a)–(1c), the generalized water flow formula (2), the momentum conservation equation (5), the effective stress principle (7), the entropy increase equation (8a)–(8c)–(10a), and (10b), and the constitutive model of the mechanical field form a THM coupling model for saturated soils. The constitutive model in this paper does not need the concepts such as yield surface and flow rule. It contains the following three parts: (1) hyperelastic relation, (11), (12a), and (12b); (2) evolution laws of elastic and nonelastic strains, (17)–(19a), and (19b); (3) “granular entropy increase” equation, (20). If the strain rate *d*_*t*_ *ε*_*ij*_ is given, the granular entropy temperature *T*_*g*_ can be determined using (20) and the elastic strain value can be obtained from (17)–(19a) and (19b). Thus, the effective stress can be calculated by (11), (12a), and (12b). Using this constitutive model, many important mechanical features of soils can be simulated, such as the hysteresis behavior of soils under cyclic shear loadings, as shown in Figure 1. In the following, this model will be applied to the analysis of nonisothermal consolidation, which is a kind of THM coupling behavior.

## 3. Simulation and Discussion

 Based on the model presented before, in this section, the nonisothermal consolidation for silty clay under cyclic thermal loadings will be simulated in order to validate the model. The elemental measured results of nonisothermal consolidation for silty clay provided by Bai and Su [11] will be used in this paper.

### 3.1. Simulation Paths

**Figure 2:**
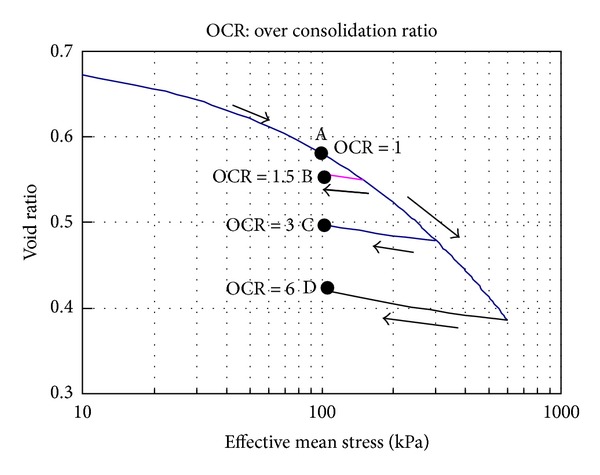
Mechanical consolidation simulation results before nonisothermal consolidation.

**Figure 3:**
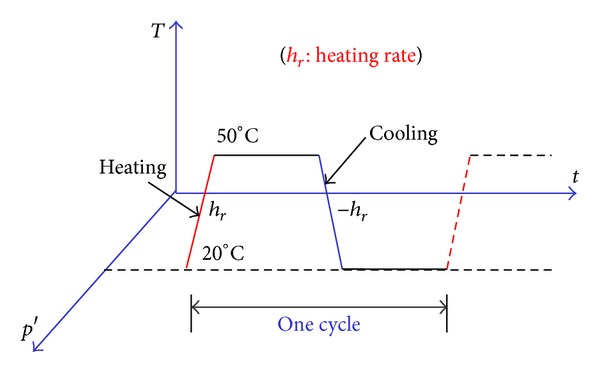
Repeated nonisothermal consolidation paths.

 As shown in Figure 2, before the nonisothermal consolidation, the clay samples are isotropically consolidated to different initial states: (1) normally consolidated state (point A in Figure 2) and (2) overconsolidated state (points B, C, and D in Figure 2). When the mechanical consolidation is finished, keeping the confining pressure constant, repeated thermal loading is applied to the clay samples, as shown in Figure 3. In each thermal loading cycle, under undrained condition, the samples are heated at a constant heating rate *h*_*r*_ to a maximum temperature and cooled at a cooling rate −*h*_*r*_ to the initial temperature. After the heating and cooling processes are finished, under drained condition, the temperature is kept constant for a period of time so that the pore pressure induced by the thermal loadings can fully dissipate.

 For isotropic consolidated state, *ε*_*s*_^*e*^ = 0. Thus, from (11), (12a), and (12b), the effective mean stress *p*′ and the confining pressure $\overset{-}{\sigma }=\sigma_{kk}/3$ are, respectively,

(21a) p′=0.6B(εve+c)0.5(εve)2+0.8B(εve+c)1.5εve +3KbβT(T−T0),

(21b) $$\begin{array}{c} \overset{-}{\sigma }=p^{\prime }+p. \end{array}$$

**Figure 4:**
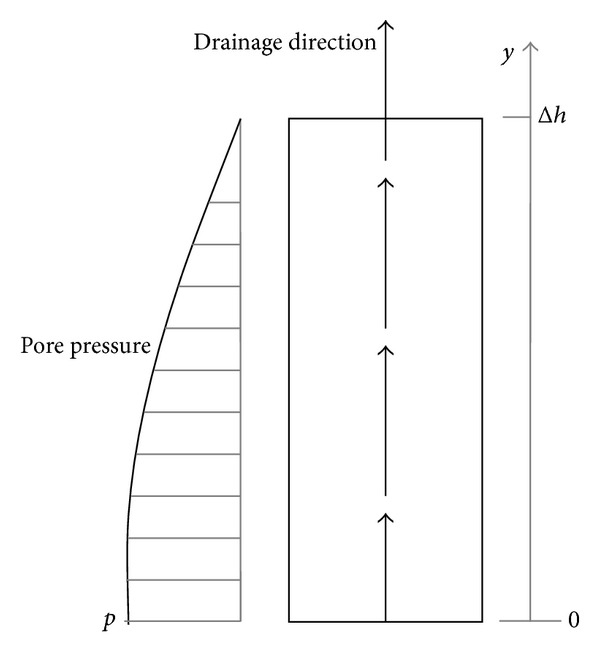
Schematic diagram of drainage in a sample.

In (21b), the Biot coefficient *α* is set at 1. The clay samples are assumed to be with uniform deformation and temperature in order to perform calculations at the elemental scale. Thus, the gradient of fluid density in (1b) and the gradient of temperature in (2) are ignored. An example of uniform upward drainage is shown in Figure 4, with a unit height of Δ*h*, a pore water pressure of zero at the top, and a pore water pressure of *p*(*y* = 0) at the bottom, provided that the pore pressure in the samples is uniform along the horizontal direction and the change rate of pore pressure along the height of the samples is zero at the bottom. Thus, the pore pressure along the height of the samples and the term [*ϕ*_fw_(*v*_*k*_^fw^ − *v*_*k*_^*s*^)]_,*k*_ in (1b) can be simplified as

(22a) $$\begin{array}{c} p\left(y\right)=-\frac{p\left(y=0\right)}{\Delta h^{2}}y^{2}+p\left(y=0\right), \end{array}$$

(22b) $$\begin{array}{c} {\left[\phi_{\text{fw}}\left(v_{k}^{\text{fw}}-v_{k}^{s}\right)\right]}_{,k}=-\frac{k}{\mu }\nabla_{k}\left(\nabla_{k}p\right)=2\frac{k}{\mu \Delta h^{2}}p\left(y=0\right). \end{array}$$

Due to uniform deformation being assumed within the samples, the deformation state at the bottom of the samples is used to represent the overall deformation state. In the following, the pore pressure *p* represents the pore pressure value at the bottom.

In the simulations, the change rates of the temperature and the confining pressure are controlled by tests; that is, *d*_*t*_(*p*′ + *p*) = *f*_1_(*t*) and *d*_*t*_ *T* = *f*_2_(*t*) are known conditions. In the mechanical consolidation, *f*_2_(*t*) = 0; in the nonisothermal consolidation, *f*_1_(*t*) = 0. Using these known conditions, the mass conservation equations (1a)–(1c), (3a), (3b), (4a), (4b), (22a), (22b), (17)–(21a), and (21b), the pore pressure development, and the strain evolution during the mechanical consolidation and nonisothermal consolidation can be calculated. Because both the total stress state and the temperature are known, the momentum conservation equation and the entropy increase equation are not used in the simulations. The main model parameters used in this paper are listed in Table 1.

### 3.2. Simulation Results

**Figure 5:**
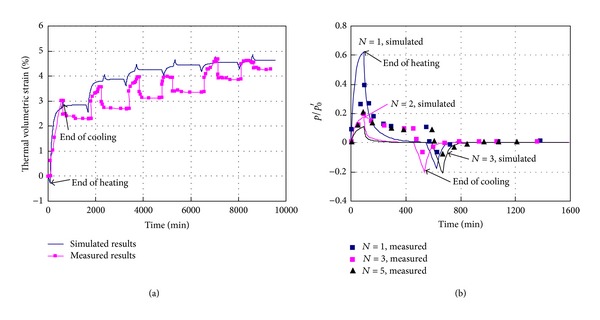
Responses of thermal volumetric strain (a) and pore pressure (b) during the repeated nonisothermal consolidation (OCR = 1; $\overset{-}{\sigma }=100\text{ kPa}$; *N* represents the cycle number; measured results are from [11]).

**Figure 6:**
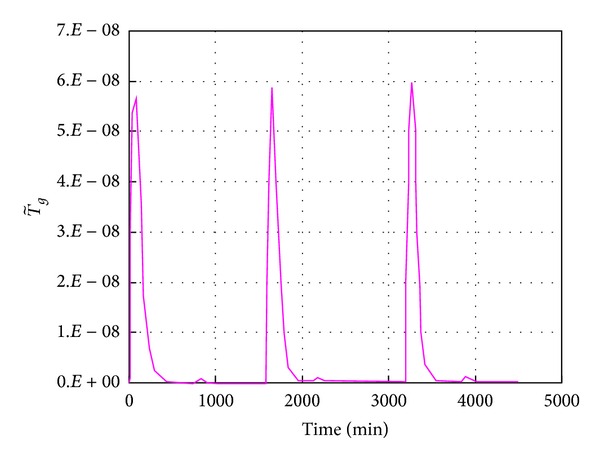
Simulation results of the granular entropy temperature evolution under repeated thermal loadings.

 The mechanical consolidation simulation results have been shown in Figure 2 and the simulation results of nonisothermal consolidation will be discussed in this section.  Figure 5 shows the responses of thermal volumetric strain and pore pressure during the repeated nonisothermal consolidation for normally consolidated silty clay.  Figure 6 shows the evolution of granular entropy temperature during the repeated nonisothermal consolidation. In the simulations, the initial temperature is 20°C, the maximum temperature is 50°C, and the heating rate *h*_*r*_ = 0.27°C/min. From Figures 5 and 6, in the undrained heating processes, the pore pressure and the granular entropy temperature increase significantly. After the heating process is finished, the pore pressure begins to dissipate and thermal volumetric deformation develops significantly until the pore pressure and the granular entropy temperature decrease to zero. The negative pore pressure is generated in the undrained cooling process, followed by a water-absorbing process and a volume expansion after the cooling process is finished.  Figure 5 shows that the nonisothermal consolidation is an unrecoverable process. With the increase of cycle number, the thermal volumetric strain is accumulated gradually. After four cycles, the volume shrinkage induced by the heating process basically equals the volume expansion induced by the cooling process. Thus, the accumulation of thermal volumetric strain disappears after certain cycles of thermal loading. Correspondingly, the maximum pore pressure in each cycle decreases gradually, while no significant change in the minimum pore pressure is observed in all cycles. This feature is attributed to the unrecoverable energy processes stimulated by the thermal loading, that is, the granular fluctuation and the triggered transient elasticity dissipation described by (18a), (18b), (19a), (19b), and (20). The simulation results shown in Figure 5 are basically consistent with the measured results provided by Bai and Su [11]. However, residual pore pressure in the nonisothermal consolidation is observed in the measured results. This may be due to the failure in considering the effect of temperature on the readings of pore pressure sensors in the experiments.

**Figure 7:**
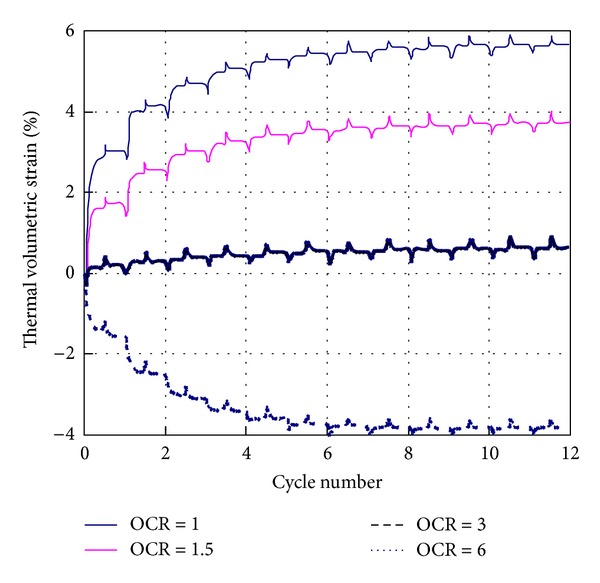
Thermal volumetric strain responses of silty clays with different OCR values under repeated thermal loadings.

Figure 7 shows the simulation results of nonisothermal consolidation under repeated thermal loadings for silty clays with different OCR values. Obviously, the nonisothermal consolidation is heavily OCR dependent. For normally consolidated or slightly overconsolidated clay, the accumulation of volume shrinkage is observed under repeated thermal loadings. On the contrary, for heavily overconsolidated clays, the accumulation of volume expansion will be generated under repeated thermal loadings. This OCR dependency of nonisothermal consolidation has been proved in many experimental studies [5–7, 11].

 In summary, the THM coupling model presented in this paper is effective for the simulations of nonisothermal consolidation, which shows obvious OCR dependency and unrecoverability. In the model, the nonisothermal consolidation is described by the granular fluctuation stimulated by the conversion process between the bound water and free water phases during the thermal loadings; see (15b) and (15e). As long as the granular fluctuation begins, the nonelastic deformation will develop due to the transient elasticity of soils. That is the physical mechanism of the nonisothermal consolidation. The ability of the model to represent the features of nonisothermal behavior of saturated soils discussed before is very important and useful for engineering areas like the shallow geothermal engineering, in which repeated thermal loadings are applied perennially, possibly resulting in the development of excess pore pressure and the significant unrecoverable deformation of the ground.

## 4. Conclusions

 A THM coupling model based on nonequilibrium thermodynamics has been established in this paper, including a constitutive model of the mechanical field without such concepts as yield surface and flow rule. The dependency of the permeability and the density of each phase on the deformation and temperature are taken into account. The entropy increase equation, in which the energy dissipation is described by the concepts of dissipative force and dissipative flow in nonequilibrium thermodynamics, is introduced as a basic governing equation of the model. In the model, the effective stress is defined as the function of the elastic strain and the dry density through the elastic potential energy density function, which considers the cohesion and thermoelastic coupling effect of soils. On the other hand, the nonelastic deformation evolution is determined by two important dissipation mechanisms called transient elasticity and granular fluctuation, which is described by the concept of granular entropy. 

 In the model, the granular fluctuation is linked with the conversion process between the bound water and free water phases under thermal loadings. Therefore, this model is able to represent the nonisothermal consolidation of saturated soils under repeated thermal loadings. Simulation results show that the nonisothermal consolidation is heavily OCR dependent and unrecoverable. Under repeated thermal loadings, volume shrinkage will be generated for normally consolidated or slightly overconsolidated clay, while volume expansion will develop for heavily overconsolidated clay. The residual thermal volumetric strain is gradually accumulated until a maximum value is reached after several cycles of thermal loading. These simulation results are consistent with the experimental results of the nonisothermal consolidation.

## Figures and Tables

**Table 1:** List of the main parameters used in this paper.

Parameter type	Elastic potential energy density function parameters			Migration coefficient		Hysteric parameters	Non-isothermal consolidation	Thermal expansion coefficient (°C^−1^)	
	*B*_0_/Pa	*B*_1_/(m^3^/kg)	*c*/[—]	*m*_1_/[—]	*m*_2_/min⁡^−(1−2*a*)/*a*^	h/[—]	*α*_*bf*_/°C^−1^	*β*_*s*_	*β*_*f*_
	*c*′/[—]	**ξ**/[—]		*m*_3_/[—]	*m*_ 4_/(kg/m^3^/min)	w/[—]	*m*_ 5_/(kg·min/m^4^)		
Silty clay	510	0.0116	0.02	0.558	1.79 × 10^3^	0.022	0.02	1 × 10^−6^	3 × 10^−4^
	0.12	0.276		0.447	48.4	0.98	3.2 × 10^−6^		

Note: the two upper and lower values correspond to the upper and lower parameters for the corresponding clay. The parameter *a* in (18b) is taken as 0.455; the initial value of porosity of bound water is 0.1. Parameters B_0_, B_1_, c, c′, and ξ can be seen in (12a) and (12b); parameters *m*_1_, *m*_2_, *m*_3_, *m*_4_, and *m*_5_ can be seen in (20); parameters *h* and *w* can be seen in (19b); parameter *α*_*bf*_ can be seen in Section 2.2.1 and (15e). In (3) and (4), *K*_*s*_ = 10^12^ Pa, *c*_*f*_ = 10^−10^ Pa^−1^, *k*_0_ = 6.4 × 10^−18^ m^2^, and *b*_*k*_ = 3.25.
